# Severe drug-associated colitis with Crohn’s features in setting of ixekizumab therapy for chronic plaque psoriasis

**DOI:** 10.1186/s12876-021-01936-w

**Published:** 2021-10-02

**Authors:** Xin Mu, John Fardy, Stephanie Reid, Julia Trahey

**Affiliations:** 1 Discipline of Medicine, Memorial University of Newfoundland, 300 Prince Philip Drive, St. John’s, NL A1B 3V6 Canada; 2 Discipline of Laboratory Medicine, Health Sciences Centre, Memorial University of Newfoundland, St. John’s, Canada; 3 Faculty of Medicine, Memorial University of Newfoundland, St. John’s, Canada

**Keywords:** Ixekizumab, Inflammatory bowel disease, Drug-associated colitis, Psoriasis, Case report

## Abstract

**Background:**

Ixekizumab is monoclonal antibody targeted against interleukin-17 (IL-17) and has been approved for use in chronic plaque psoriasis. Despite its efficacy in treating psoriasis, concerns have been raised regarding Ixekizumab’s potential to induce and exacerbate inflammatory bowel disease (IBD).

**Case presentation:**

Here we report the new onset of severe drug-associated colitis with surgical complications in a 45-year-old male patient who was receiving Ixekizumab therapy for chronic plaque psoriasis. Review of the patient’s colonic pathology demonstrated acute inflammatory changes with features of Crohn’s disease. The patient remained disease-free 9-months following his hospitalization and cessation of Ixekizumab.

**Conclusions:**

This case raises suspicion for an association between Ixekizumab and IBD and calls on clinicians to have heightened awareness of potential risks before prescribing anti-IL-17 agents.

## Background

Interleukin-17 (IL-17) is a proinflammatory cytokine that plays a complex role in T-cell-mediated immunity and has emerged as a key target in the biologic therapy of various autoimmune diseases [1, 2]. In psoriasis, it is postulated that IL-17 synergizes with other cytokines to upregulate the expression of pro-proliferative genes leading to cutaneous inflammation and dermal hyperplasia [1]. Ixekizumab, a monoclonal antibody with IL-17 antagonism, has been shown to be efficacious in the treatment of chronic plaque psoriasis and specifically, in those with moderate to severe disease [3, 4]. Despite only infrequent reports of inflammatory bowel disease (IBD) in clinical trials [4, 5], there have been increasing case reports of new onset IBD in association with Ixekizumab therapy [6–8]. Here we report a case of severe drug-associated colitis with features of Crohn’s disease complicated by toxic megacolon and bowel perforation requiring surgical management in context of Ixekizumab exposure for chronic plaque psoriasis.

## Case presentation

A 45-year-old male patient presented to our institution with a 3-week history of diffuse abdominal pain, tenesmus, and over 10 episodes of non-bloody diarrhea per day. The patient did not have nausea or vomiting nor did he report fever, chills, or unintentional weight loss. In the week prior to his presentation, he was assessed by his family physician who had ordered a panel of stool nucleic acid amplification tests which were negative for gastrointestinal infections. He was then trialed on a brief course of proton pump inhibitor as an outpatient with no improvement in his symptoms before presenting to the emergency department.

The only significant past medical history for our patient was a long-standing history of plaque psoriasis for which he was being treated with Ixekizumab. He had previously undergone multiple courses of biologic treatments with adjunctive phototherapy but did not achieve reasonable control of his disease until he was switched to Ixekizumab approximately 9 months ago. The patient did not have a family history of IBD or early onset colorectal cancer. He is a life-long non-smoker and he does not report excessive alcohol use. There was no history of recent travel, blood transfusions, intravenous drug use, or new sexual contacts.

**Fig. 1:**
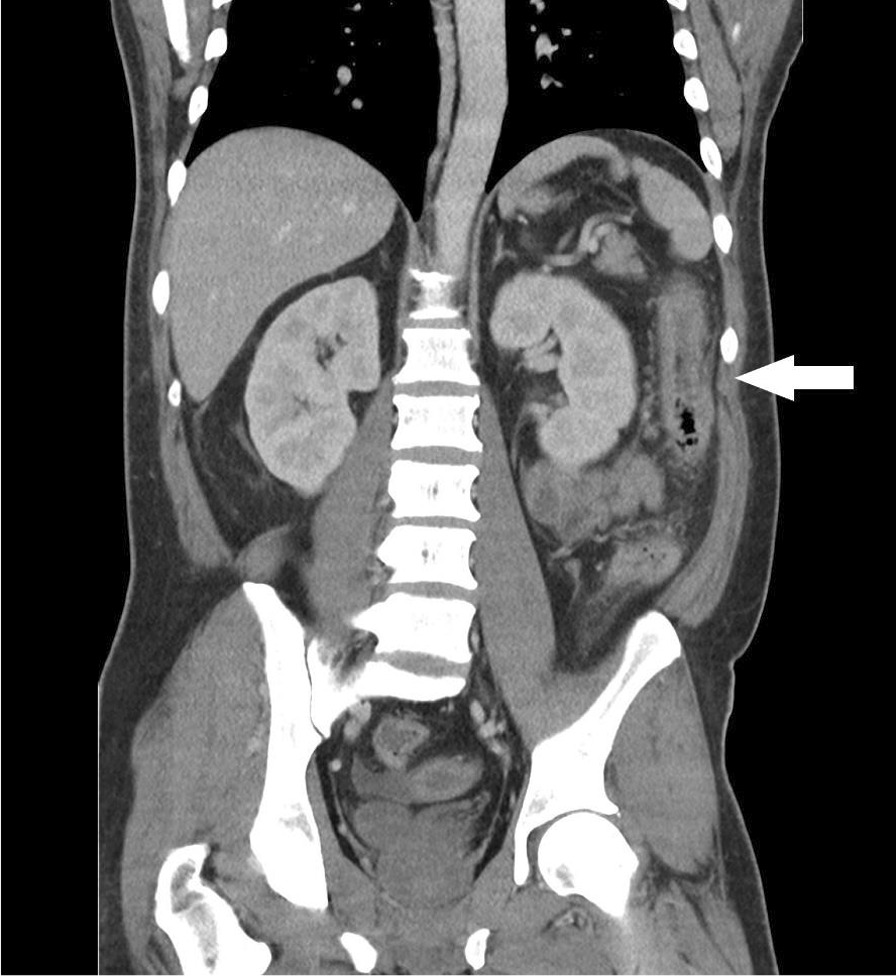
Coronal contrast-enhanced CT image shows marked circumferential wall thickening of the descending and sigmoid colon with luminal narrowing, associated mucosal hyperenhancement, as well as adjacent inflammatory stranding and edema (see arrowhead)

On initial assessment, the patient was hemodynamically stable with diffuse non-peritonitic abdominal pain on physical examination. His laboratory investigations demonstrated marked elevation in his inflammatory marker (C-reactive protein of 388 mg/L) as well as a hepatocellular pattern of liver enzyme elevation (aspartate aminotransferase 234 U/L, alanine aminotransferase 142 U/L, alkaline phosphatase 143 U/L). A computed tomography (CT) of the abdomen and pelvis was performed revealing continuous circumferential bowel wall thickening of the left colon from the rectum to the splenic flexure on a background of reactive retroperitoneal lymphadenopathy (Fig. 1). The patient was admitted to the internal medicine service and our gastroenterology experts were consulted for further endoscopic evaluation.

On flexible sigmoidoscopy, the patient was noted to have punched-out ulcerations in the left colon with overlapping regions of normal mucosa. Given that these endoscopic findings could be in keeping with IBD as well as cytomegalovirus (CMV) colitis, the patient was started empirically on oral Mesalamine and IV Ganciclovir while awaiting the results from his endoscopic biopsy. Interestingly, the biopsy showed no viral cytopathic changes and CMV immunohistochemical staining was negative. The specimen did however have non-specific changes of cryptitis, epithelioid granulomas and mixed inflammatory infiltrates with areas of fibrinoid necrosis. Following discussions with our gastroenterology and pathology experts, Ganciclovir was discontinued and high dose corticosteroid therapy was initiated for probable drug-associated colitis in context of his Ixekizumab exposure.

**Fig. 2:**
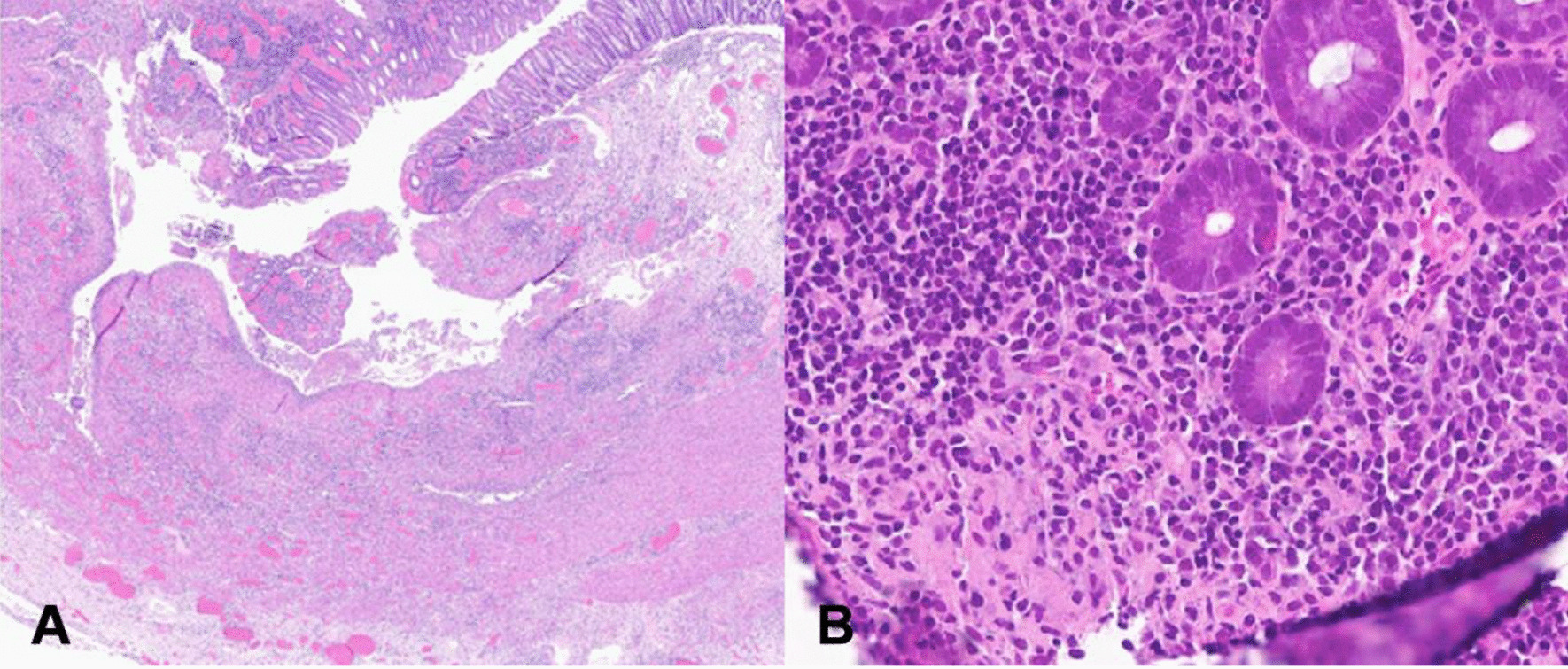
**a** Examination on low power view of resected colon specimen show areas of transmural inflammation, fissure formation, and ulceration. **b** High power view of mucosal biopsy specimens demonstrates granuloma formation and active inflammatory changes

Despite initial clinical response to steroid therapy, the patient developed toxic megacolon 2 days later and was complicated by perforated viscus as evident from the presence of intraperitoneal free air on abdominal X-ray. Following an emergent total colectomy, the patient was transferred to the intensive care unit (ICU) for continued support. During his week-long ICU admission, the patient suffered from persistent intra-abdominal sepsis requiring high dose vasopressors which prompted repeated exploratory laparotomies revealing ongoing small bowel ischemia. The patient had further resection of his distal ileum and was successfully weaned from vasopressors before undergoing an end ileostomy. He continued rehabilitation for an additional 2 weeks on the surgical ward before being discharged home with follow-ups to our gastroenterology and general surgery specialists. At 9-months post-discharge, the patient had been progressing well with no recurrence of clinical disease in the absence of any immunosuppressive therapy. Further review of the patient’s surgical and endoscopic specimens revealed the presence of epithelioid histocytes and widespread fissuring ulcerations with intermittent regions of transmural inflammation favouring a Crohn’s-like pattern (Fig. 2). Despite features of Crohn’s on pathology, the patient’s unremarkable family history, lack of recurrence disease following surgical interventions, and atypical clinical presentations [9] collectively raise suspicions for drug-associated colitis in setting of the patient’s Ixekizumab therapy.

## Discussion and conclusions

The precise role of IL-17 in IBD pathophysiology remains unclear. Traditionally, it is believed that interleukin-23 (IL-23) regulates the secretion of IL-17 by T helper type 17 cells (Th17) which promote the release of pro-inflammatory mediators leading to subsequent intestinal mucosal damage [10]. However, preclinical studies in animal models have been conflicting in determining whether IL-17 is truly exacerbating or protective against IBD [11]. While IL-23 inhibition has been shown to be an effective mechanism of IBD therapy in clinical trials, IL-17 inhibition has not yielded any evidence of benefit but rather, has been associated with an increase in adverse events [12, 13]. Experts postulate these results may be a reflection of the heterogenous functions of various IL-17 subtypes; some of which may serve as pro-inflammatory mediators while others are protective against IBD [12]. In any case, these results highlight the complex nature of cytokine homeostasis in the gut mucosa and its dysregulations in IBD manifestations.

Psoriatic diseases have been associated with IBD in addition to many other gastrointestinal pathologies including celiac disease, acid reflux, and irritable bowel syndrome (IBS) [14]. The underlying pathophysiology that link psoriasis with IBD is complex and not well-understood. However, aberrations of innate immune response, including Th17 activities, seem to play a prominent role in both conditions’ pathogenesis [15]. Unlike in IBD, anti-IL-17 biologics have produced much more consistent benefits as therapeutics in psoriasis [16]; suggesting different mechanisms of inflammatory injury between the 2 diseases. Given the intricacy of innate immunity and variations of cytokine pleiotropy in different tissues, it would not be surprising that inhibition of 1 cytokine may have opposing effects on 2 associated conditions.

Ixekizumab is 1 of 3 available anti-IL-17 biologics that have been approved for treatment of psoriasis by the United States Food and Drugs Administration. In the initial efficacy trials examining the use of Ixekizumab for plaque psoriasis, new presentations of IBD were not found to be a frequent adverse effect in the experimental groups [4]. Similarly, trials evaluating other anti-IL-17 agents also reported only rare instances of IBD manifestations [17, 18]. Despite increasing reports of inflammatory bowel pathologies in those undergoing anti-IL-17 treatment, recent studies examining the incidence of IBD among psoriatic patients did not find a significant increase in the risk of IBD development with exposure to IL-17 antagonist [5, 19, 20]. While these studies emphasize that de novo IBD in setting of IL-17 inhibition is rare, reassurance should be viewed cautiously in light of the low number of outcome events including the presence of many zero-event trials. Further, the collection of IBD history in several studies were only documented on a volunteered basis; raising concern that the true IBD incidence may in fact be underreported. In any case, additional studies are needed to better characterize the role that IL-17 inhibition has in IBD pathophysiology.

Anti-IL-17 agents—such as Ixekizumab—are becoming increasingly prevalent in their use against psoriatic diseases. Although their precise actions in the gut mucosa remain unclear, our case raises concern that IL-17 inhibitors may be complicit in the induction of inflammatory bowel pathologies. Physicians should be vigilant in their use of anti-IL-17 therapy and patients on active treatment should be monitored closely for the development of gastrointestinal symptoms.

## Data Availability

Not applicable.

## Abbreviations

CMV: Cytomegalovirus
CT: Computed tomography
IBD: Inflammatory bowel disease
ICU: Intensive care unit
IL-17: Interleukin-17
IL-23: Interleukin-23
Th17: T helper type 17 cells
